# Pulmonary Metabolism of Substrates for Key Drug-Metabolizing Enzymes by Human Alveolar Type II Cells, Human and Rat Lung Microsomes, and the Isolated Perfused Rat Lung Model

**DOI:** 10.3390/pharmaceutics12020117

**Published:** 2020-02-01

**Authors:** Katarina Rubin, Pär Ewing, Erica Bäckström, Anna Abrahamsson, Britta Bonn, Satoshi Kamata, Ken Grime

**Affiliations:** 1 Drug Metabolism and Pharmacokinetics, Research and Early Development, Respiratory, Inflammation and Autoimmune (RIA), BioPharmaceuticals R&D, AstraZeneca, 431 83 Gothenburg, Swedenpar.ewing@astrazeneca.com (P.E.); erica.backstrom@astrazeneca.com (E.B.); annaabrahamsson@hotmail.se (A.A.); britta.bonn@astrazeneca.com (B.B.); 2 Department of Thoracic Surgery, Institute of Development, Aging, and Cancer, Tohoku University Graduate School of Medicine, Sendai 980-0000, Japan; a2m1035j@yahoo.co.jp

**Keywords:** drug metabolizing enzymes, lung metabolism, pulmonary drug delivery

## Abstract

Significant pulmonary metabolism of inhaled drugs could have drug safety implications or influence pharmacological effectiveness. To study this in vitro, lung microsomes or S9 are often employed. Here, we have determined if rat and human lung microsomes are fit for purpose or whether it is better to use specific cells where drug-metabolizing enzymes are concentrated, such as alveolar type II (ATII) cells. Activities for major hepatic and pulmonary human drug-metabolizing enzymes are assessed and the data contextualized towards an in vivo setting using an ex vivo isolated perfused rat lung model. Very low rates of metabolism are observed in incubations with human ATII cells when compared to isolated hepatocytes and fewer of the substrates are found to be metabolized when compared to human lung microsomal incubations. Reactions selective for flavin-containing monooxygenases (FMOs), CYP1B1, CYP2C9, CYP2J2, and CYP3A4 all show significant rates in human lung microsomal incubations, but all activities are higher when rat lung microsomes are used. The work also demonstrates that a lung microsomal intrinsic clearance value towards the lower limit of detection for this parameter (3 µL/min/mg protein) results in a very low level of pulmonary metabolic clearance during the absorption period, for a drug dosed into the lung in vivo.

## 1. Introduction

Inhaled drugs are commonly used in the treatment of patients with respiratory diseases. The inhalation route aims to provide efficient delivery of drugs, resulting in a high local concentration while keeping the systemic levels low [1]. In theory, significant metabolism in the lung could have drug safety implications or influence pharmacological effectiveness either through the lowering of local parent drug concentration or the production of active metabolites. Most xenobiotics are metabolized by phase I (oxidative) enzymes such as cytochrome P450 (CYP), flavin-containing monooxygenases (FMOs), monoamine oxidase (MOA), xanthine oxidase/aldehyde oxidase (XO/AO) and epoxide hydrolase (EH) [2], with CYP being the most important family of enzymes, accounting for about 75% of the total human drug metabolism [3]. The liver is the major site of drug metabolism in the body, but the role of other tissues such as the lung, kidney, and gastrointestinal tract should not be ignored [4]. Indeed, studies have revealed the presence (mRNA expression or protein) of CYP1A1, CYP1B1, CYP2A6, CYP2A13, CYP2B6, CYP2C8/18, CYP2D6, CYP2E1, CYP2F1, CYP2J2, CYP2S1, CYP3A4/5, CYP3A43, CYP4B1, EHs, and FMOs as the major drug-metabolizing enzymes present in different pulmonary cells, including alveolar type I (ATI) and type II (ATII) cells, Clara cells, ciliated columnar epithelial cells, and macrophages [5,6,7]. Nevertheless, the expression of drug-metabolizing enzymes in lung tissue is known to be much lower than that of the liver and it has been estimated that CYP-dependent drug metabolic activity may be less than 10% of that found in the liver [5,8]. 

Unlike the liver, where a single cell type (hepatocyte) makes up 90% of the volume of the organ and accounts for almost all drug-metabolizing capacity, the complexity of the lung, which has over 40 cell types differing in morphology and function and very intricate architecture [9], makes the study of drug metabolism challenging. Perhaps as a result of this complexity, the differential bronchial and pulmonary blood supply to the central and peripheral lung and the fact that drugs may reach higher concentrations in epithelial and sub-epithelial tissue after inhalation compared to systemic or oral dosing [10] has meant that in vitro pulmonary drug metabolism data have never been used to calculate and predict in vivo drug clearance by the whole organ, in contrast to this common practice for hepatic clearance [11]. This presents a problem to pharmacokinetic researchers: What relevance should be put on in vitro lung intrinsic clearance data? If metabolism is observed in vitro, what does that translate to in vivo? The aim of this work was to determine the functional activity of all the major hepatic and pulmonary human drug-metabolizing enzymes using different in vitro systems and to qualitatively contextualize the data towards an in vivo setting using an ex vivo isolated perfused lung model. 

Microsomes are typically employed to study phase I drug metabolism. However, as stated above, at the cellular level the lung is much more heterogeneous than the liver and consequently microsomes prepared from whole lung tissue may dilute out the drug-metabolizing activity concentrated in a few cell types. Since human ATII cells contain many drug-metabolizing enzymes [12], these cells are used here to study functional drug metabolism and compare it to whole human lung microsomes and human hepatocytes. This is done to determine if lung microsomes offer an acceptable system for the study of pulmonary drug metabolism and to contextualize the data against the principal drug-metabolizing cell of the body. To allow an assessment of how in vitro data scales to in vivo qualitatively, rat lung microsomes are used in conjunction with an ex vivo isolated perfused rat lung model.

The drug-metabolizing enzymes studied were chosen because of their possible importance to pulmonary drug metabolism (CYPs 1B1, 2C19, and 2J2) [12] or based on their being of primary importance in human hepatic metabolism (FMO and CYPs 1A2, 2B6, 2C8, 2C9, 2C19, 2D6, 2E1, and 3A4/5) with appropriately selective reactions previously defined [13,14,15,16,17]. 

## 2. Materials and Methods

### 2.1. Chemicals

Albendazole, 1-OH-albendazole, benzydamine, phenacetin, acetaminophen, bufuralol, flutamide, diclofenac, and paclitaxel were obtained from the AstraZeneca compound library. 2-OH-bupropion, 1-OH-bufuralol, 1-OH-midazolam, 4-OH-midazolam, 4-OH-mephenytoin, 4-OH-diclofenac, 2-OH-flutamide, benzydamine N-oxide, 4-nitrocatechol, β-Nicotinamide adenine dinucleotide hydrate (NADPH) and DMSO were obtained from Sigma (St. Louis, MO, USA). Bupropion was obtained from Kemprotec Limited (Smailthorn, Carnforth, United Kingdom), midazolam from Lipomed (Cambridge, MA, USA), 6α-OH-paclitaxel from EMD Chemicals (San Diego, CA, USA), and S-mephenytoin from Toronto Research Chemicals (Toronto, ON, Canada). P-nitrophenol and potassium dihydrogen phosphate were obtained from Thermo Fisher Scientific (Gothenburg, Sweden) and dipotassium hydrogen phosphate and magnesium chloride were obtained from Merck (Kenilworth, NJ, USA). Formic acid (98–100%, analytical grade) was obtained from Merck (Darmstadt, Germany), acetonitrile from Rathburn (Walkerburn, UK), and ethanol from CCS Healthcare AB, Borlänge, Sweden. Deionized water was produced in an ELGA purification system (High Wycombe, UK). 

### 2.2. Compound Selection

The set of substrates selective for human drug-metabolizing enzymes which were used are as follows: phenacetin (CYP1A2), albendazole (CYP2J2), flutamide (CYP1B1), bupropion (CYP2B6), paclitaxel (CYP2C8), diclofenac (CYP2C9), S-mephenytoin (CYP2C19), bufuralol (CYP2D6), P-nitrophenol (CYP2E1), midazolam (CYP3A4/5), and benzydamine (FMO). A list of which test systems were used to study the metabolism of these substrates and the enzyme-specific metabolites is presented in Table 1.

### 2.3. Human Hepatocytes

Primary human hepatocytes from three single donors (a 67-year-old male, a 64-year-old female, and a 42-year-old female) obtained from Kaly-Cell (Plobsheim, France) were used in the present study. 

### 2.4. Patients and Human Lung Tissue Preparation

Resected human lung tissues were obtained from patients who underwent lung resection for primary lung cancer at the Department of Thoracic Surgery at Tohoku University Hospital or at Japanese Red Cross Ishinomaki Hospital. Small (1–2 g) lung tissues (without any pathological abnormalities including tumors, overt inflammation, and fibrosis) were immediately immersed in a tissue preserve solution (Stem Survive; Kurabo, Osaka, Japan) and used to isolate cells within 6 h after surgery. The Ethics Committees at Tohoku University School of Medicine and at Japanese Red Cross Ishinomaki Hospital approved this study and the experiments conformed to the principles set out in the World Medical Association Declaration of Helsinki (2017-1-352). All subjects gave their informed consent. 

### 2.5. Isolation of ATII Cells from Human Lungs 

ATII cells were isolated from human lung tissues as previously described [18]. A live/single cell gated CD45-negative/EpCAM-high/T1α-low/VE-cadherin-negative cell subset was sorted as an ATII cell population by a FACS Aria II cell Sorter and FACS Diva version 6.1 (BD Biosciences, San Jose, CA, USA). The following antibodies were used to obtain single-cell suspensions: Alexa Fluor 700-mouse anti-human CD45 antibody (clone HI30; Biolegend, San Diego, CA, USA), phycoerythrin-mouse anti-human EpCAM antibody (clone 1B7; eBioscience, San Diego, CA, USA), Alexa Fluor 647-rat anti-human T1α antibody (clone NC-08; Biolegend), and fluorescein isothiocyanate-mouse anti-human VE-cadherin antibody (clone 55-7H1; BD Pharmingen, San Diego, CA, USA). To eliminate cells 7-amino actinomycin D (eBiosciences) was used. This routinely resulted in more than 90% of prosurfactant protein-C-positive ATII cells.

### 2.6. Incubation of Drug-Metabolizing Enzyme Substrates with Rat and Human Lung Microsomes

Lung microsomes from a pool of 219 untreated male Sprague Dawley rats and human lung microsomes (pool of two male and two females, all Caucasian non-smokers, 12 to 66 years old) were obtained from XenoTech (Brussels, Belgium). Prior to incubation, individual stock solutions were prepared separately for each substrate as follows: bupropion and midazolam, 4 mM in methanol; bufuralol and flutamide, 4 mM in acetonitrile/water (50:50, *v/v*); benzydamine and S-mephenytoin, 2 mM in acetonitrile/water (50:50, *v/v*); albendazole, 0.2 mM in DMSO/ethanol/water (50:38:12, *v/v*/*v*); diclofenac, 0.4 mM in water; p-nitro phenol, 1 mM in acetonitrile/water (50:50, *v/v*), phenacetin, 1 mM in ethanol; and paclitaxel, 1 mM in methanol. Aliquots were diluted in incubation buffer to give the following incubation concentrations: bupropion 23 µM, midazolam 4 µM, bufuralol 23 µM, flutamide 18 µM, benzydamine 15 µM, albendazole 2 µM, S-mephenytoin 20 µM, p-nitro phenol 14 µM, paclitaxel 8.3 µM, phenacetin 18 µM, and diclofenac 5.3 µM. 

All reactions contained individual, not pooled, substrates, and were performed in glass vials in total volumes of 400 μL. The incubation mixtures consisted of potassium phosphate buffer (0.1 M, pH 7.4 at 37 °C) containing magnesium chloride (5 mM) and rat or human lung microsomes at two concentrations (1 mg/mL and 2 mg/mL). After 5 min preincubation at 37 °C, the reaction was initiated by addition of NADPH (incubation concentration of 1 mM). The reactions were terminated at 0, 5, 10, 20, and 45, or 60 min by removing aliquots (50 µL) into acetonitrile (180 µL) containing formic acid (0.2%, *v/v*) and an analytical internal standard. After vortex mixing and centrifugation (3226*g*, 20 min) in an Eppendorf 5810R centrifuge, the supernatants were diluted (1:1, *v/v*) with water containing formic acid (0.2%, *v/v*) to match the initial mobile phase and analyzed for parent substrates and metabolites using an ultra-performance liquid chromatography tandem mass spectrometry system (UPLC-MS/MS) as described below. The activity of the enzymes was calculated from the initial slope of metabolite formation over time. 

### 2.7. Incubation of Drug-Metabolizing Enzyme Substrates with Isolated Human ATII Cells and Human Hepatocytes

ATII cells and human hepatocytes were used at identical concentrations. Prior to incubation a single stock solution containing 4 Mm flutamide, bupropion, bufuralol, albendazole, midazolam, and benzydamine was prepared in DMSO. An aliquot of this stock solution (3 µL) was added to a culture plate containing ATII cells or hepatocyte suspension (3 mL, 0.33 million cells/mL), resulting in a final incubation concentration of 4 µM. The incubations were allowed to continue for 2 and 4 h before termination by removal of the incubation media (3 mL) from the plates into tubes containing acetonitrile (9 mL). The samples were centrifuged (3226 *g*, 4 °C, 20 min) before an aliquot (10 mL) of each supernatant was transferred to a separate vial. Finally, the supernatants were diluted with water (1:1, *v/v*) and analyzed for parent substrates and metabolites using an UPLC quadropole time of flight system (UPLC-QToF) as described below.

### 2.8. Assessment of Drug Metabolism in the Ex Vivo Isolated Perfused Rat Lung (IPRL)

The IPRL method was used as previously described by Ewing et al. [19]. In brief, rats were euthanized using an overdose of pentobarbital. Lung and heart were dissected en bloc and the pulmonary artery was catheterized. A plastic tube was fitted in the trachea and the lung was placed in an artificial thorax. Hydrostatic pressure was used to perfuse pulmonary circulation with an albumin Krebs-Ringer buffer at neutral pH. Isolated lung preparation was ventilated using a rodent ventilator (No. 7025, Ugo Basile, Comerio, Italy) at 75 breaths per minute and a tidal volume on average of 1.5 mL. IPRL preparations were routinely screened for any sign of pulmonary edema by visual inspection and accordingly accepted or rejected for use in studies. Experiments were perfused in a single-pass mode, prohibiting re-circulation of perfusate in the lung. 

Benzydamine (4 mM) was solubilized in a phosphate buffer containing ethanol (1%, *v/v*). Albendazole (4 mM) was delivered as drug suspension in saline (0.1% Tween, and pH was adjusted to pH 5 using citric acid). Both substrates were dosed to IPRL via nebulization of drug formulation (1 mL) and the nebulizer was run to dryness. Aerosolization was achieved using a mesh nebulizer (eFlow™, Pari, Starnberg, Germany). In brief, a closed respiratory circuit was devised to allow drug administration to the IPRL. Inspiratory airflow produced by IPRL allowed inhalation and deposition of aqueous aerosols in the peripheral lung. The targeted lung deposited dose was 20 nmol per lung, corresponding approximately to a lung deposited dose of 20 µg/kg bodyweight. Following inhalation, perfusate exiting the lung was collected using a sample collector. Perfusate was collected up to 1 h following drug administration at the time points 0, 1, 2, 3, 4, 6, 8, 10, 12, 18, 22, 28, 33, 40, 50, and 60 min, and aliquots (50 µL) were retained for bioanalysis. At the end of the experiment, the lungs were weighed and frozen prior to sample preparation and analysis. 

### 2.9. Bioanalysis

The lungs were homogenized in a Ringer solution using bead-beating technology (Bertin technologies, Montigny le Bretonneux, France). For the analysis, isolated perfused rat lung homogenate (50 µL), lung perfusate (50 µL), and microsomal suspension (50 µL) were protein precipitated by the addition of acetonitrile (180 µL) containing formic acid (0.2%, *v/v*), and an analytical internal standard. After vortex mixing and centrifugation (3226g, 20 min, 4 °C) the supernatants were diluted (1:1, *v/v*) with water containing formic acid (0.2%, *v/v*) to match the initial mobile phase. The samples were then injected into a UPLC-MS/MS system (Agilent 6490, Agilent Technologies Inc., Wilmington, Delaware) and analyzed under a positive or negative ionization mode. The mobile phases were (A) water and 0.2% formic acid and (B) 100% acetonitrile and 0.2% formic acid. Separation was achieved by applying a gradient elution on a Zorbax Eclipse Plus C18 column (2.1 × 50 mm, 1.8 µm, Agilent Technologies Inc.). The fragmentor voltage used in all UPLC-MS/MS analytical methods was 380 V. The gradients for 1-OH-albendazole and 2-OH-bupropion, given as percentages of mobile phase B, were 0 to 0.30 min, 5%; 0.30 to 6.00 min, 70%; 6.00 to 7.00 min, 95%; 7.00 to 7.50 min, 95%; and 7.50 to 7.51 min, 5%. The gradient for 1-OH midazolam and 4-OH midazolam started at 5% with an increase to 50% in 1.70 min; 1.70 to 2.30 min, 95%; and 2.30 to 2.31 min, 5%. For the other metabolites the gradient started at 5% with an increase to 95% in 1.50 min; 1.50 to 2.30 min, 95%; and 2.30 to 2.31 min, 5%. The gradient was linear between each stated time point (see Table 2 for UPLC-MS/MS parameters). The hepatocyte and ATII cell samples were analyzed using a UPLC-QToF (Agilent 6560 IM, Agilent Technologies Inc.) system and the samples were analyzed in both positive and negative ionization mode, with a mobile phase of (A) 0.1% formic acid and (B) 100% acetonitrile. Separation was achieved by applying a gradient elution on an Acquity UPLC C18 column (2.1 × 100 mm, 1.7 µm, Waters, Etten-Leur, Netherlands), starting with 5% B with a linear increase to 70% B in 6.00 min, after which B was immediately increased to 90% and retained to 6.70 min. Quantitation of metabolite formed was performed using external standard curves prepared with authentic metabolite standards. Quantification was carried out with Agilent MassHunter software. 

## 3. Results

### 3.1. Incubation of Drug-Metabolizing Enzyme Substrates with Rat and Human Lung Microsomes

**Figure 1:**
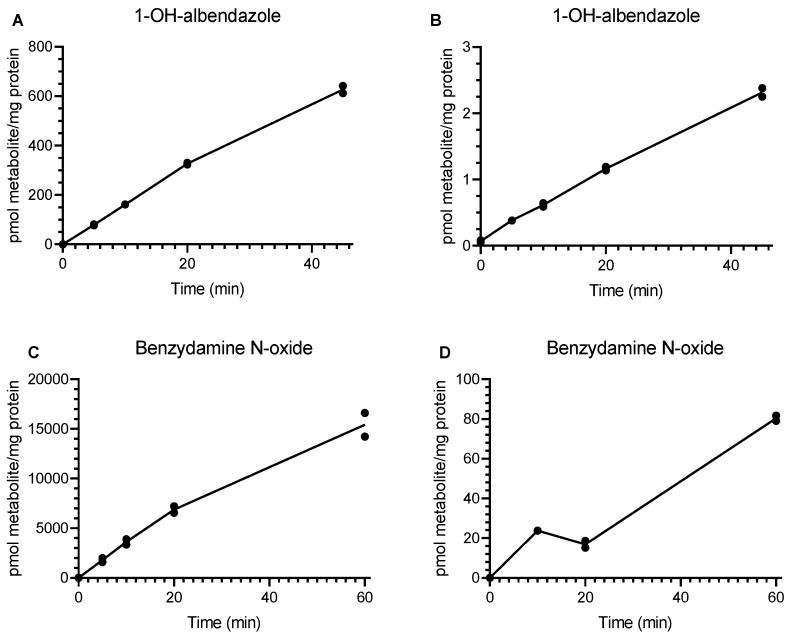
Rate of metabolite formation after incubation of drug-metabolizing enzyme substrates with rat (**A**,**C**) and human (**B**,**D**) lung microsomes: (**A**,**B**) CYP2J2-dependent 1-OH-albendazole; (**C**,**D**) FMO-dependent benzydamine N-oxide.

Eleven enzyme-selective substrates were incubated with rat and human lung microsomes. Enzyme activities (rates of metabolite formation, pmol/min/mg microsomal protein) are presented in Figure 1a–d and Table 3. The highest enzyme activity was FMO-dependent benzydamine N-oxidation in rat and human lung microsomes, but the activity of rat lung FMO was found to be over 200-fold higher than that in human. Albendazole hydroxylation, 4-nitrocatechol, and bupropion hydroxylation all had similar enzyme activities in rat lung microsomes. The corresponding activities for CYP2J2, CYP2E1, and CYP2B6 in human lung microsomes were all very low (undetectable for CYP2B6 and CYP2E1 and 300-fold lower activity for CYP2J2-dependent albendazole hydroxylation in human compared to rat). CYP3A4-dependent midazolam 1-hydroxylation, CYP2C9-dependent diclofenac 4-hydroxylation, and CYP1B1-dependent flutamide 2-hydroxylation were all detectable in human lung microsomal incubations, but were observed to be four-fold, three-fold, and nine-fold lower than the corresponding rat activity, respectively. CYP2C8-dependent paclitaxel hydroxylation and CYP2C19-dependent mephenytoin hydroxylation were undetectable in rat and human lung microsomes.

### 3.2. Analysis of Drug-Metabolizing Enzyme Activity in Isolated Human ATII Cells and Human Hepatocytes

The rates of metabolite formation (pmol/10^6^ cells) after 2 and 4 h of incubation in the two systems are shown in Table 4. For ATII cells, the highest rates of metabolism were for CYP2J2-dependent albendazole hydroxylation and FMO-dependent benzydamine N-oxidation. The rates of midazolam 4-hydroxylation (CYP3A4), flutamide 2-hydroxylation (CYP1B1), and bufuralol 1-hydroxylation (CYP2D6) were approximately 10-fold lower than for the FMO and CYP2J2 substrates with ATII cells. Isolated human hepatocytes metabolized all the substrates at much higher rates than ATII cells, with CYP2J2-dependent albendazole hydroxylation and FMO-dependent benzydamine N-oxidation being, respectively, 10-fold and 30-fold higher in hepatocytes.

### 3.3. IPRL

**Figure 2:**
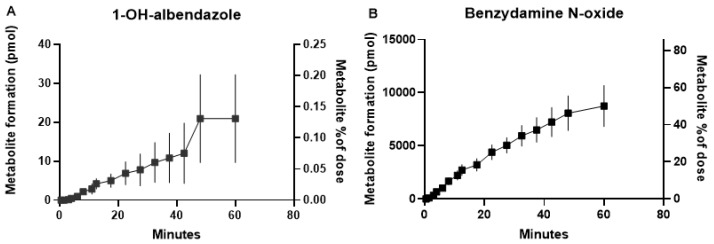
Metabolite formation, from the drug-metabolizing enzyme-selective substrates albendazole (**A**) and benzydamine (**B**), following nebulized administration of approximately 20 µg/kg bodyweight or 20 nmoles per lung to isolated perfused rat lung.

Albendazole (16 ± 0.6 nmoles) and benzydamine (19 ± 3.8 nmoles) were dosed to IPRL, with amounts of formed 1-OH-albendazole and benzydamine N-oxide quantified. The percentage metabolism was calculated from the ratio of the amount of metabolite (nmoles) to the amount of parent dosed (nmoles). The data were plotted against time post dose (Figure 2).

## 4. Discussion

The drug-metabolizing capacity of the lungs is generally thought to be substantially lower than that of the liver. This assumption is based on lower expression levels of the drug-metabolizing enzymes primarily responsible for the clearance of most therapeutic drugs and the fact that there is little evidence of significant extra-hepatic metabolism contributing to the systemic clearance of these drugs [7,11,12]. It is, however, conceivable that inhaled drugs may be subject to local pulmonary drug metabolism due to the potentially high concentrations of doses achieved in the vicinity of the relevant lung enzymes after dosing. This is considered a rare and minimal risk since many inhaled small molecule drugs have near-complete systemic blood bioavailability [20]. Nevertheless, prior to clinical drug development it is important to have available in vitro systems that can be used with confidence to determine if a novel inhaled candidate drug has the possibility of being metabolized in the lungs of humans and/or pre-clinical species used in drug safety testing. Sub-cellular fractions of lung tissue are typically used for such in vitro experiments [21] and our experience is that observing metabolism of novel inhaled candidate drugs in these studies is extremely rare, unless the molecule is designed as a pro-drug [22]. One purpose of this work was to determine if these in vitro systems are fit for purpose or whether it is more appropriate to use specific cells where drug-metabolizing enzymes are concentrated. Here, we used isolated human ATII cells, since these cells contain many drug-metabolizing enzymes [12], and compared the results with human lung microsomal data. A second aim of this work was to establish the relevance of any observed in vitro metabolism. In essence the question was, if metabolism is observed or not observed, what does this mean in a whole organ context when drugs access the drug-metabolizing enzymes as an inhaled formulation? For this we used isolated perfused lung preparations and two of the most rapidly metabolized substrates in vitro (albendazole and benzydamine). Because it was not possible to readily obtain whole human lungs to perform perfused organ experiments, rat lung was used. In order to make this in vitro–ex vivo whole organ extrapolation relevant in the context of human drug metabolism, rat and human lung microsomal rates of metabolism were compared. 

Since the work aimed to study the importance of human drug-metabolizing enzymes, with rat primarily being used to assess the significance of the in vitro data in a whole organ context, selective substrates for each human drug-metabolizing enzyme were chosen based on previously published works [13,14,15,16,17]. For those substrates where metabolism was observed following incubations with human lung microsomes (benzydamine, albendazole, flutamide, midazolam, and diclofenac), the rat enzymes involved in metabolizing benzydamine, midazolam, and diclofenac had been previously determined and shown to correspond well to their human counterparts [23,24]. 

Human lung microsomes exhibited very low rates of activity for all the tested drug-metabolizing enzymes when compared to previously determined human liver microsomal activities [13,14,15,16,17]. When selected substrates were incubated with isolated human hepatocytes and ATII cells, very low rates of metabolism were again observed in the lung cells in comparison to the liver cells. With the ATII incubations, only albendazole and benzydamine gave detectable rates of metabolism (Table 3 and Table 4). Activities were higher for rat lung microsomes than for human lung microsomes, indicating that the isolated perfused rat lung model could provide insight into in vitro to in vivo extrapolation of drug metabolism in a whole organ setting. The model was used in single pass mode rather than recycling the perfusate. Each data point in Figure 2 therefore represents the extent to which the parent drug was metabolized as it was absorbed from the airway to perfusate. The data indicate that for both albendazole and benzydamine the absorption process continued over the course of the experiment with approximately 50% of benzydamine converted to the N-oxide metabolite, compared to less than 0.2% of the albendazole dose being metabolized. For pre-clinical work with potential candidate drugs, intrinsic clearance (CL_int_) of the parent molecule is typically determined for liver microsomes, hepatocytes, or lung microsomes when investigating metabolic stability. The commonly recognized method for calculating clearance is the dose divided by the area under the drug concentration–time curve (dose/AUC). However, this is simply the integral of the rate of clearance/concentration of the drug, and, hence, if a molecule is metabolized with a rate of 1 nmole/min/mg microsomal protein and the drug concentration is 1 µM (1 nmole/mL), the CL_int_ will be 1000 µL/min/mg protein. For 1-OH-albendazole the rate of rat lung microsomal metabolism was found to be 0.016 nmoles/min/mg protein and the incubation concentration was 2 µM; hence, the CL_int_ can be calculated to be 0.016/2 = 8 µL/min/mg microsomal protein. Typically, the lower limit of significance for determination of a CL_int_ value is around 3 µL/min/mg protein for a 1 mg microsomal protein/mL incubation, with this representing an around two-fold change in the parent molecule LC-MS chromatogram peak area over a 60 min incubation (the upper end of incubation time for which microsomes remain viable). Thus, what we have demonstrated here is that an in vitro CL_int_ value approximately three times greater than the lower quantifiable limit of 3 µL/min/mg lung microsomal protein may give rise to a very low rate of pulmonary metabolism (0.1–0.2% of the dose, corresponding to a rate of metabolism in the IPRL of 0.3 pmoles/min) in rat lung after inhaled administration. A CL_int_ value of 3 µL/min/mg protein would therefore translate to an extremely low level of dose metabolized during the inhalation period. This simple analysis is likely to also be relevant for human lung metabolism in vitro and in vivo. We have noted here, as others have previously [25], that human lung metabolism in vitro appears to be lower than that for rat. As such, if there is any doubt over a candidate drug’s metabolic stability in human lungs after inhaled dosing, an isolated perfused rat lung experiment, in addition to rat and human lung microsomal or S9 fraction incubations, should provide sufficient tools with which to investigate the question. 

The FMO substrate benzydamine was the most extensively metabolized substrate by rat and human lung microsomes, with rat appearing to have much greater FMO activity than human, confirming a previous report [25]. Additionally, as Table 4 and an earlier work [15] show, the hepatic rate of metabolism is considerably higher than pulmonary. Using the calculation method detailed above, the rat lung microsomal CL_int_ value was approximately 23 µL/min/mg lung microsomal protein, and this translated into an IPRL rate of metabolism of 250 pmoles/min with roughly 50% of the dose metabolized in one hour.

## 5. Conclusions

In summary, the data gathered in this work indicate that isolated ATII cells do not offer more than lung microsomes for studying pulmonary drug metabolism and that FMO and CYPs 1B1, 2C9, 2J2, and 3A4 all appear to be active in human lung microsomal preparations. Additionally, rat appears to have greater drug-metabolizing activity than human lung. Finally, and perhaps most importantly, this work has demonstrated that a lung microsomal intrinsic clearance value towards the lower limit of detection for this parameter (3 µL/min/mg protein) can still result in metabolic clearance for a drug dosed into the lung in vivo, albeit at a very low level. 

## Figures and Tables

**Table 1:** List of substrates and the in vitro and/or ex vivo systems used to study metabolism in the present study. Legend: ATII, alveolar type II; FMO, flavin-containing monooxygenases; IPRL, isolated perfused rat lung.

Substrate	Test System	Enzyme	Metabolites
Albendazole	Microsomes, ATII, hepatocytes, IPRL	CYP2J2	1-OH-albendazole
Phenacetin	Microsomes	CYP1A2	Acetaminophen
Benzydamine	Microsomes, ATII, hepatocytes, IPRL	FMO	Benzydamine N-oxide
Bufuralol	Microsomes, ATII, hepatocytes	CYP2D6	1-OH-bufuralol
Bupropion	Microsomes, ATII, hepatocytes	CYP2B6	2-OH-Bupropion
P-nitrophenol	Microsomes	CYP2E1	4-nitrocatechol
Flutamide	Microsomes, ATII, hepatocytes	CYP1B1	2-OH-flutamide
Midazolam	Microsomes, ATII, hepatocytes	CYP3A4/5	1-OH-midazolam 4-OH-midazolam
Paclitaxel	Microsomes	CYP2C8	1-OH-paclitaxel
Diclofenac	Microsomes	CYP2C9	4-OH-diclofenac
S-mephenytoin	Microsomes	CYP2C19	4-OH-mephenytoin

**Table 2:** Ultra-performance liquid chromatography tandem mass spectrometry system (UPLC-MS/MS) parameters for metabolites of the drug-metabolizing enzyme selective substrates. Legend: CE, collision energy; Rt, retention time; LLOQ, lower limit of quantification.

Metabolite	Transition	CE (volts)	Rt (min)	LLOQ (nM)
Acetaminophen	152.1 > 110.0	23	0.73	3.7
2-OH-flutamide	291.1 > 205.1	18	1.5	0.07
2-OH-bupropion	256.1 > 238.0	14	2.6	1.5
6α-OH-paclitaxel	870.0 > 104.9	80	1.5	3.3
4-OH-diclofenac	312.0 > 230.0	42	1.4	0.05
4-OH-mephenytoin	235.1 > 149.9	22	1.0	4.4
1-OH-bufuralol	278.2 > 185.9	18	0.88	0.06
1-OH-albendazole	282.1 > 250.1	18	2.4	0.03
4-nitrocatechol	154.0 > 124.0	14	1.0	0.2
1-OH-midazolam	342.1 > 203.1	34	1.6	0.4
4-OH-midazolam	342.1 > 325.1	26	1.5	0.08
Benzydamine N-oxide	326.2 > 84.0	38	1.2	4.6

**Table 3:** Rat and human lung microsomal rates of metabolite formation for selected CYP and FMO substrates. Legend: n/a, not applicable (below limit of quantification).

Metabolite Formed	Rat Lung Microsomes (pmol/min/mg Protein)	Human Lung Microsomes (pmol/min/mg Protein)
OH-albendazole	16.5	0.053
	16.2	0.055
Benzydamine N-oxide	358	1.36
	329	1.39
4-nitrocatechol	14.9	n/a
	14.6	
2-OH-bupropion	19.7	n/a
	10.8	
1-OH-bufuralol	0.089	n/a
	0.102	
4-OH-midazolam	1.79	n/a
	1.26	
1-OH-midazolam	0.447	0.103
	0.363	0.089
2-OH-flutamide	0.933	0.099
	0.897	0.123
4-OH-diclofenac *	0.102	0.033
Acetaminophen	5.20	n/a
	5.53	

* One replicate.

**Table 4:** Rate of metabolite formation, (pmol/million cells) measured by UPLC quadropole time of flight system (UPLC-QToF) after incubation of drug-metabolizing enzyme substrates with human ATII cells (ten individual donors) and human hepatocytes (four individual donors). Legend: n/a, not applicable (below limit of quantification of 1 nM); n/d, not determined.

Sample ID	Time (h)	1-OH-albendazole	Benzydamine N-oxide	1-OH-bufuralol	2-OH-bupropion	2-OH-flutamide	4-OH midazolam
ATII cells							
15-017	2	78	75	n/a	n/d	n/a	n/a
15-017	4	93	120	n/a	n/d	n/a	n/a
15-018	2	87	93	n/a	n/d	n/a	n/a
15-018	4	123	138	n/a	n/d	n/a	n/a
15-019	2	102	75	n/a	n/d	n/a	n/a
15-019	4	177	135	n/a	n/d	n/a	n/a
15-020	2	75	114	9	n/d	9	6
15-020	4	90	159	9	n/d	18	9
15-022	2	75	129	n/a	n/d	n/a	9
15-022	4	90	141	n/a	n/d	n/a	n/a
Hepatocyte							
S1193T-1	2	766	3024	1697	1318	3769	300
S1193T-1	4	931	4817	2477	1649	4721	336
S1193T-2	2	724	2694	1339	1111	3360	270
S1193T-2	4	826	4084	1799	1300	3928	282

## Abbreviations

ATI	alveolar type I
ATI	alveolar type I
ATII	alveolar type II
CL_int_	intrinsic clearance
CYP	cytochrome P450
BLOQ	below limit of quantification
EH	epoxide hydrolase
FMO	flavin-containing monooxygenases
IPRL	isolated perfused rat lung
MOA	monoamine oxidase
MS/MS	tandem mass spectrometry
PAH	polycyclic aromatic hydrocarbons
QToF	quadropole time of flight
UPLC	Ultra-performance liquid chromatography
XO/AO	xanthine oxidase/aldehyde oxidase
